# Chronic non-communicable diseases in Cameroon - burden, determinants and current policies

**DOI:** 10.1186/1744-8603-7-44

**Published:** 2011-11-23

**Authors:** Justin B Echouffo-Tcheugui, Andre P Kengne

**Affiliations:** 1 Hubert Department of Global Health, Rollins School of Public Health, Emory University, Atlanta, Georgia, USA; 2 Medical Research Council of South Africa, University of Cape Town, South Africa

**Keywords:** chronic diseases, Cameroon, burden, determinants, policies

## Abstract

Cameroon is experiencing an increase in the burden of chronic non-communicable diseases (NCDs), which accounted for 43% of all deaths in 2002. This article reviews the published literature to critically evaluate the evidence on the frequency, determinants and consequences of NCDs in Cameroon, and to identify research, intervention and policy gaps. The rising trends in NCDs have been documented for hypertension and diabetes, with a 2-5 and a 10-fold increase in their respective prevalence between 1994 and 2003. Magnitudes are much higher in urban settings, where increasing prevalence of overweight/obesity (by 54-82%) was observed over the same period. These changes largely result from the adoption of unfavorable eating habits, physical inactivity, and a probable increasing tobacco use. These behavioral changes are driven by the economic development and social mobility, which are part of the epidemiologic transition. There is still a dearth of information on chronic respiratory diseases and cancers, as well as on all NDCs and related risk factors in children and adolescents. More nationally representative data is needed to tract risk factors and consequences of NCDs. These conditions are increasingly been recognized as a priority, mainly through locally generated evidence. Thus, national-level prevention and control programs for chronic diseases (mainly diabetes and hypertension) have been established. However, the monitoring and evaluation of these programs is necessary. Budgetary allocations data by the ministry of health would be helpful, to evaluate the investment in NCDs prevention and control. Establishing more effective national-level tobacco control measures and food policies, as well as campaigns to promote healthy diets, physical activity and tobacco cessation would probably contribute to reducing the burden of NCDs.

## Background

Cameroon is a low-income country with a rapidly increasing population, which was estimated at 19.088 million individuals in 2008 [1]. The country is undergoing social and economic changes, which are resulting in increased urbanization with a potentially negative impact on health-related behaviors. Recent figures suggest that the economy of the country has been growing, with an average annual growth of 3% from 2000 to 2009 [2]. Growth in trade has also increased, such that imports and exports are valued at 56% of GDP [2]. The country's gross national income per capita was US$2,180 in 2008 [1]. The wealth generated by the economic growth is unequally distributed as reflected by the Gini coefficient of 0.446 in 2001 and the fact that at least 32.8% of the population lives below the poverty line (< US$1 per day) [1]. Such disparities may have health consequences. Increasing urbanization is exposing the Cameroonian population to highly processed foods (usually high in fat, salt, and sugar) and increasingly sedentary lifestyles. Currently, 57.6% individuals live in urban areas, a population that grew at a rate of 3.90% per year from 2005 to 2010 [1].

As a consequence of the aforementioned socio-economic changes, Cameroon may now be experiencing the double burden of infectious and chronic non-communicable diseases (NCDs). The burden of infectious diseases is largely driven by malaria, HIV/AIDS and tuberculosis. In 2007, the prevalence of HIV among adults aged 15 to 49 years, was in the order of 5.1% [1]. Malaria caused approximately 116 deaths per 100,000 people [1]. The prevalence and incidence of tuberculosis were estimated to be respectively 150 and 190 per 100,000 population in 2008 [1]. Yet at the same time, the country experiences an increase in the burden of NCDs, displaying elements of a health transition, in which a mix of both acute and chronic diseases now coexist in the same population and compete for limited resources [3].

This article examines the current burden of NCDs and their determinants in Cameroon, as well as the local actions undertaken to tackle these conditions.

## Methods

We searched PubMed up to February 2011 for studies addressing chronic non-communicable diseases in Cameroon, using a combination of the following keywords: "diabetes", "hypertension ", "obesity", "physical activity ", "diet", "nutrition", "cancers", "asthma" "sickle-cell disease", "chronic disease", "chronic disease intervention", "policy", "urbanization/urban/rural/migration", "smoking" and "Cameroon". We did not limit by date or language. We hand-searched the reference lists of articles identified. We also examined published peer-reviewed reports and reviews, as well as book chapters. We used publications from the World Health Organization (WHO), International Diabetes Federation (IDF), World Bank, United Nations (UN), Food and Agriculture Organization (FAO), International Agency for Research on Cancer (IARC) and we accessed their websites for relevant information. The eligible publications were scrutinized to extract data on the frequency of major chronic diseases (cardiovascular diseases, diabetes, chronic respiratory diseases and cancers), their risk factors (individual and societal) and complications. We also retrieved information on key health systems features and local policies initiated to address NCDs. Using data retrieved from various types of studies across a broad range of pathophysiology, public health, and psychosocial literature, we seek to provide a synthesis of the most up-to-date, relevant, and key literature regarding NCDs in Cameroon.

### Burden of chronic non-communicable diseases

In Cameroon, chronic diseases (including cardiovascular diseases, diabetes, respiratory diseases, and cancers) accounted for 848.1 deaths per 100,000 in 2002, corresponding to 43% of all deaths (and 1,480 DALYs per 1,000 - 21% of total DALYs), whereas 56% of all deaths were related to infectious diseases [4]. Chronic diseases are now emerging in both rural and urban areas of Cameroon, but are particularly prominent in urban areas. However, little is known about the distribution of chronic disease among socio-economic strata of the society, especially in urban areas.

### Chronic non-communicable diseases

#### Cardiovascular diseases

A number of studies, mostly cross-sectional have quantified the burden of hypertension at the community level. Fezeu et al collated data from some of these studies conducted on the same site (in the city of Yaoundé) at different time points, to describe the temporal variation in blood pressure levels and prevalence of hypertension in Cameroon based on contemporary diagnostic criteria [5]. Between 1994 and 2003, there was a rightward shift of cumulative distribution curves of systolic and diastolic blood pressures, and the prevalence of hypertension increased by 2- to 5-fold in rural and urban Cameroonian men and women [5]. More specifically, the age-standardized prevalence of hypertension changed from 20.1% to 37.2% among women and from 24.4% to 39.6% among men. Over a ten-year period, systolic (SBP) and diastolic (DBP) blood pressure levels significantly increased in rural women (SBP +18.2 mmHg, DBP +11.9 mmHg) and men (SBP +18.8 mmHg, DBP +11.6 mmHg). In urban areas, SBP increased in women (+8.1 mmHg) and men (+6.5 mmHg), and DBP increased only in women. In a much recent, larger and representative survey of adults urban dwellers of the most populated city of Cameroon (Douala), Kengne et al found a prevalence of hypertension of 20.8% among adults [6].

#### Diabetes mellitus

One of the earliest elaborated community-based study on the burden of diabetes in Cameroon dates back to 1994. In this survey, the age-standardized prevalence of diabetes in the rural and urban population ranged from 0.8% to 1.6% among adults Cameroonians [7]. In 1997-1998, the reported the prevalence rate for diabetes mellitus across rural and urban areas ranged from 2.9% to 6.2% [8]. Over a 10-year period (1994-2003) there was an almost 10-fold increase in diabetes prevalence in Cameroonian adults [9,10]. In 2010, the International Diabetes Federation (IDF) estimated the nation prevalence of diabetes among adults aged 20 to 79 years at 4.4% [11]. Prevalent undiagnosed diabetes is also very high - about 80% [9,11]. Furthermore, glycemic control in known diabetes patients is often very poor; only about one in four known diabetic patients in a population-based survey had optimal fasting blood glucose levels [10].

#### Chronic respiratory diseases

There is a lack of national prevalence data on chronic respiratory diseases in Cameroon. This may be partly related to the difficulties in the use of spirometry, the gold standard for chronic respiratory obstructive disease (COPD) diagnosis. In 1997-1998, a study estimated the age-standardized prevalence of wheeze, self-reported asthma, and asthma care via cross-sectional representative surveys among adults and children (5-15 years) in urban and rural populations from Cameroon [12]. The prevalence of self-reported wheeze was 1.3% to 2.5% in adults, and 0.8% to 5.4% in children. There were no consistent patterns of urban-rural prevalence. Peak flow rates varied with age, peaking at 25-34 years, and were higher in urban areas (age adjusted difference 22-70 L/min). Awareness (83%-86% versus 52%-58%) and treatment (43%-71% versus 30%-44%) of asthma was higher among those with current wheeze in rural areas. Use of inhaled drugs, particularly steroids, was rare.

#### Cancers

In Cameroon, there is a dearth of national data on the frequency of and trends in cancers. A number of hospital-based studies have described features of cancers, mostly gynecological or uro-genital [13-16]. However, it is difficult to rely on estimates from these studies, which were small in size and probably not representative of the whole country. We therefore relied on estimates from the IARC databases. In 2008, IARC estimated that population-based age-standardized incidence of cancers for both sexes was 92.1 per 100,000 persons per year in Cameron, and the risk of receiving a diagnosis of cancer before the age of 75 was 8.7% [17]. The age-standardized rate of cancer deaths was 73.1 per 100,000 persons per year and the risk of dying of a cancer before the age of 75 was 11% [17]. For both sexes, the five most common cancers are breast, uterine cervix, liver, non-Hodgkin lymphoma and prostate cancers. Prostate cancer is the most common malignancy in men, with an age-standardized incidence and mortality rates of 19.2 and 15.2 per 100, 000 persons per year, respectively. Breast and cervical cancers are the most prevalent tumors in women; the age-standardized incidence and mortality rates are 27.9 and 16.6 per 100,000 persons per year for breast cancer, and 24 and 19 per 100000 persons per year for cervical cancer [17]. The relatively high frequency of cervical cancer in Cameroon has been attributed to the high prevalence of human papillomavirus [18,19]. However, no data exist to substantiate this claim.

Cervical, breast and prostate cancer screening programs have been implemented [20-22]. It is unclear whether these programs have contributed to lowering cancers-related deaths over time. There is no national population-based cancer registry; however, a registry covering the capital city (Yaoundé) has been described [23].

### Risk Factors

#### Obesity

In 2003, a large survey of adults aged ≥ 15 years in four main Cameroonian towns (Yaoundé, Douala, Garoua and Bamenda), found that more than more than 25% of urban men and almost half of urban women were either overweight or obese, with 6.5% of men and 19.5% of women being obese [24]. In this study, the prevalence of obesity showed variation with age in both genders. The body mass index (BMI) based prevalence of obesity was higher in men (6.5%) than that based on waist-to-hip ratio (3.2%). Among women, using waist-to-hip ratio and waist circumference yielded the highest prevalence of obesity (28%) and BMI the lowest (19.5%). The prevalence of obesity increased with the level of education (duration of education) in both sexes. In terms of trends, between 1994 and 2003, the age-standardized prevalence of BMI ≥ 25 kg/m^2 ^increased significantly in the rural area (+54% for women and +82% for men), while the prevalence of central obesity (WC ≥ 80 cm [women], ≥ 94 cm [men]) increased significantly only in the urban population (+32% for women and +190% for men) [25].

#### Physical activity

An early study of physical activity levels in Cameroon found an inverse correlation between physical activity and BMI in urban men and women, rural men, but not women [8]. A subsequent and more recent study showed a significantly lower objectively measured free living physical activity energy expenditure (PAEE) in urban dwellers than in rural dwellers (44.2 vs.59.6 kJ/kg/day, p < 0.001) and a higher prevalence of the metabolic syndrome (17.7 vs. 3.5%, P < 0.001)[26]. In this study, each unit increase in PAEE (kJ/kg/day) was associated with a 2.1% lower risk of prevalent metabolic syndrome. The population attributable fraction of prevalent metabolic syndrome due to being in the lowest quartile of PAEE was estimated at 26.3% (25.3% in women and 35.7% in men) indicating that modest population-wide changes in PAEE may have significant benefits in terms of reducing the emerging burden of metabolic diseases in Cameroon. Also, objectively measured PAEE was inversely associated with levels of blood glucose independently of adiposity, fitness in both urban and rural Cameroonians [27]. Although these three studies strongly suggest that the prevalence of inactivity in Cameroon is increasing, especially in urban areas, representative studies providing population-based or nation-wide data on levels of physical activity or sedentary time are lacking.

#### Tobacco

There is a scarcity of data on the prevalence of smoking in Cameroon. Unpublished data from a national survey indicate that the overall prevalence of smoking is approximately 6.4% in Cameroon, with a higher frequency in male (8.2%) than in females (1.0%) [28]. There are no data on the trends in smoking prevalence over time. However, based on the rising number of tobacco multinational companies in the country, it is not unreasonable to think that tobacco consumption is probably increasing.

#### Diet

Nation-wide data on dietary intake and patterns are not available in Cameroon. However, a small-scale study comparing a central urban (cosmopolitan) area and a rural one indicated that the intake of energy, fat and alcohol was higher in rural men and women than in urban subjects [29]. In rural women, the intake of carbohydrates and protein was also higher. In this study, eight of the 10 foods eaten in the highest amount and contributing most to energy intake differed between the rural and urban population. These contradicting results were explained at the time by the probable much higher physical activity levels in rural areas. Regarding the intake of sodium, in 1991-1994 the total salt intake in Cameroon was reported to be less than 100 mmol/day. However, these data obtained using a non-nationally representative sample and sub-optimal methods, needs updating [30]. The trends in sodium intake may have changed with time. Moreover, the contribution of specific foods of the Cameroonian diet on the intake of sodium is unknown.

In the absence of nationally representative survey data, we used indirect data from the Food and Agricultural Organization (FAO) food balance sheets on food availability, to crudely measure trends in dietary intake in Cameroon. Over 40 years, per capita dietary energy intake has increased from 2,001 kilocalories per day in 1962 to 2,269 kilocalories in 2007. During the same period, fat consumption increased from 26 g to 43 g per day [31].

#### Alcohol consumption

Alcohol consumption is relatively high in Cameroon, with the percentage of life-time abstainers estimated at 11% in male and 18% in females, in 2008[28].

#### Dyslipidemia

The dyslipidemia profile of Cameroonian is unknown. To date there are no published studies on the national prevalence of dyslipidemia/hypercholesterolemia in Cameroon.

### Children

Children are an understudied group for chronic diseases, thus very little known about the risk factors and burden of chronic diseases in this group. In 2010, the total number of prevalent cases of type 1 diabetes in children was about 37,500 in the African region, which includes Cameroon [11]. One cross-sectional study reported physical activity levels and nutrient intake in 12-16 years old urban Cameroonian. Boys had a lower BMI and reported higher energy expenditures and physical activity levels than girls; and 25% of these adolescents had a high fat intake [32]. Another study showed that physical activity levels among rural children were more than twice that in urban children, and activities for rural children was mostly work-related [33]. Rural children consumed more fruits and vegetables and less fat containing foods. Among urban children there was a trend toward a larger age-adjusted mean BMI [33]. However, these two studies offer a very limited picture of the impact of physical activity, and diet on chronic diseases in children, as these factors were not related to disease outcomes and because of the imprecision of the subjective assessment of physical activity (questionnaires covering a very short period-24 hours recall) [32].

In Cameroonian children with sickle cell disease, the risk of stroke may be elevated. A hospital-based study among homozygous sickle cell patients, showed a relatively high prevalence of stroke of 6.67% [34].

### Complications of chronic diseases

Except for diabetes, the complications of chronic diseases have not been extensively investigated in Cameroon. In 2010, the estimated number of deaths attributable to diabetes was 11,852 [11]. In a hospital-based study, Koki et al found a prevalence of diabetic retinopathy and macular edema of 42% and 10.6% respectively[35]. Microalbuminuria was also described in about 53.1% of patients with diabetes in a hospital-based study [36]. The prevalence of diabetes-related foot lesions, diabetic neuropathy, ischemia and food deformity were 13.0%, 27.3%, 21.3% and 17.3%, respectively in a cross-sectional hospital survey [37]. A subsequent study found a hospital-based prevalence of diabetic foot ulceration of 13% over a 8-year period (200-2007) [38]. In a hospital audit, up to 10.2% of diabetic patients admissions were related to coma, with a case fatality rate of 20% [39].

An audit in a tertiary care hospital found an 11.5% prevalence of clinical heart failure or asymptomatic left ventricular dysfunction among 1,218 patients with hypertension followed over a 10-year period (1995-2005) [40]. In this study, systolic dysfunction and isolated diastolic heart failure were found in 64% and 23% of cases, respectively. More than half (56.4%) of the patients had at least one comorbidity and 30.7% had multiple co-morbidities, which included renal impairment, overweight/obesity, COPD, gout, anemia, diabetes mellitus, atrial fibrillation, stroke, and ischemic heart disease [40]. In a 9-year prospective study, classical cardiovascular risk factors (ageing, smoking, hyperglycemia, and SBP) were significantly associated with all-cause mortality [41]. A 10 mm Hg higher SBP, a 10 year increase in age, elevated fasting capillary glucose and current smoking were associated with 23%, 29%, 19%, and 114% greater risk of death.

## Societal determinants of chronic non-communicable diseases

### Social and economic drivers

Demographic changes are key drivers of the epidemic of chronic diseases. A continuous growth of the Cameroonian population is anticipated. The proportion of the population aged 60 years or more (5% of total population in 2008) is also expected to rise by 4.6% by 2025 [1].

Urbanization and social mobility that has accompanied economic development is leading to increasing obesity, diabetes and hypertension. Studies have consistently shown an urban-rural gradient in the prevalence of hypertension, diabetes and obesity, which are higher in urban than in rural areas [5,7,8,10,25]. In a study, both lifetime exposure to an urban environment and current urban residence were independently related to diabetes. Lifetime exposure to an urban environment was strongly associated with fasting blood glucose concentration (r = 0.23; p < 0.001), with the prevalence of diabetes or impaired fasting glucose being highest for individuals who had the longest duration of urban contact [42]. When compared to their French counterparts, urban Cameroonians had higher abdominal adiposity; they also displayed a higher increase in obesity-related abnormalities compared with their rural counterparts [43]. There is also an urban-rural gradient in physical activity levels, with significantly higher activity in rural than in urban dwellers and a protective effect of physical activity on glucose intolerance. The urban-rural difference in physical activity is characterized by a rural-to-urban left shift in the population distribution of physical activity energy expenditure [26,27].

### Cultural drivers

Cameroonians are increasingly exposed to contemporary (and unhealthy) global food trends. Moreover, the habitual Cameroonian diet is heavy in starch and sugar, and probably contributes to increasing obesity rates [29]. A qualitative survey among Cameroonian adolescents showed a strong preference for sweetened foods, with a trend showing a nutrition transition over time from a traditional diet in rural areas to a more westernized diet, characterized by processed food and sweet beverages, in urban areas [32].

In most rural and some urban Cameroonian settings, health beliefs, knowledge, lay perceptions, and health behavior interact strongly to contribute to the occurrence of chronic diseases [44,45]. Owing to misconceptions indicated by popular health beliefs, many Cameroonians fail to take appropriate measures for prevention and control of diabetes and its risk factors [44,45]. Obesity is still perceived as a sign of good living, because it confers respect and influence [44]. Such lay perceptions are borne out of a contextual environment, in which most people are poor, hungry, and deprived and, therefore, view obesity as an obvious social marker for affluence [44,45]. Persisting poverty and deprivation in Cameroon means that traditional perceptions and cognitive imagery about lifestyle risk factors of diabetes are unlikely to change in any significant way, unless socio-culturally appropriate health interventions are implemented.

### Environmental drivers

There are no national figures on the health effects of environmental pollutants in Cameroon. However, sources of health-damaging pollution are likely to be indoor smoke from solids fuels, motor vehicles (mostly second hand/low quality vehicles from Europe), industries burning dirty fossil fuels (coal, fuel oil, and diesel) in appliances that generally do not have emission control devices, and domestic use of highly-polluting coal, wood (still commonly used for cooking and heating) in areas (mostly rural) without electricity coverage.

### Social and economic consequences of chronic non-communicable diseases

There is a dearth of information on the specific impact of chronic diseases on the economy, society, education and health care systems in Cameroon. In 2001, a study estimated the average direct medical cost of treating a patient with diabetes at US$489 with 56% of this cost spent on hospital admissions, 33.5% on antidiabetic drugs, 5.5% on laboratory tests and 4.5% on consultation fees. The direct medical costs for treating all diabetic patients in Cameroon represented about 3.5% of the national budget for the 2001-2002 financial year [46]. In 2010, the mean expenditure for diabetes was estimated to be US$83 per person per year [11]. The indirect and intangible costs of diabetes have not been evaluated.

## Management and prevention of chronic non-communicable diseases

Although infectious diseases (HIV/AIDS, Malaria, Tuberculosis) dominate the allocation of scarce public health resources, there is growing evidence that chronic diseases are beginning to receive government attention [47,48]. Unfortunately, it is impossible to quantify the Cameroonian government's level of investment in chronic diseases control with precision, since the health budget allocation is not always specified by disease groups and therefore no budgetary allocation data could be located.

### Health system

The bulk of health care in Cameroon is provided by the public sector. The private sector is growing, but mainly in large cities. The sector of health insurance is embryonic. The public sector health care system consists of district hospitals and community clinics/health centers at the primary level, which are largely nurse-driven other than in major urban areas. These are complemented by secondary and tertiary care hospitals, although the latter are unevenly distributed in the country, concentrated in the more developed, urbanized areas. In terms of distribution of the health workforce, there were approximately 2 physicians, and 16 nurses and midwives per 100,000 people in Cameroon for the 2000-2009 period [1].

Major deficiencies exist in both the quality and access to care such that chronic diseases and their risk factors are diagnosed infrequently and managed inadequately. Clinics are swamped by large patient numbers. Health care workers do not always have the knowledge or skills, in particular communication skills, to provide optimal patient-centred care. Stock shortages of essential drugs, lack of access to others (e.g., lipid-lowering agents), and limited recourse to testing (e.g., glycated hemoglobin) hinder health care delivery. In addition, there is a need to overcome health care provider 'inertia' related to effective communication and knowledge translation with patients [49].

### Chronic non-communicable disease control

Since 2001, a number of health policies relevant to chronic diseases management, as well as those addressing standards and norms for a primary health care package and community care workers, have been formulated and adopted by the Cameroonian Ministry of public health. The baseline Cameroon Burden of Diabetes (CAMBoD) survey provided new scientific knowledge that guided health policy and the implementation of a diabetes and hypertension program [47]. Diabetes and hypertension were recognized as emerging public-health problems, and incorporated into a national 10-year plan for health promotion, with this leading to the creation of two bodies within the Ministry of Public Health: the Department of Applied Research; and the Department of Disease Control that focuses on non-communicable diseases. This also occurred as result of an increasing political will to develop policies and national programs to prevent and control non-communicable chronic diseases [47]. The aim of the nationwide diabetes-hypertension control program is to promote equitable access to quality health services in order to reduce the morbidity and mortality linked to these conditions. The specific objectives of this program for the 2004-2010 period were: 1) To obtain 25% reduction in the prevalence of modifiable risk factors for diabetes and hypertension in the population through the Integrated Communication Plan; 2) To ensure optimal management of all people with diabetes detected in all health institutions in the country; 3) To obtain 25% reduction in complications linked to diabetes and hypertension, 4) To obtain 100% coverage of the Health Districts by the program; 5) To implement the National Diabetes-Hypertension Control Plan. The various strategies adopted to achieve the formulated objectives were - capacity building for home and health-service management of diabetes and hypertension, prevention of diabetes and hypertension, reduction of risk factors, reduction of complications of diabetes and hypertension, epidemiologic surveillance, management process, training and development operational research on diabetes and hypertension, partnerships for the control of diabetes and hypertension, reinforcement of institutional capacities [47].

Within the framework of the Cameroon Essential Non-Communicable Disease Health Invention Project (CENHIP) a number of studies have been carried out, showing that a task shifting to nurse could help to control chronic diseases [50]. Implementation of a 26-month protocol, for the care of hypertension by trained nurses, in urban and rural areas, was shown to be effective at controlling blood pressure. This led to a drop of systolic and diastolic blood pressures by 11.7 mm Hg (95% confidence interval (CI): 8.9-14.4) and 7.8 (95% CI: 5.9-9.6), respectively (p < 0.001) between baseline and final visits [38]. A similar program for diabetes, led to a drop of mean fasting capillary blood glucose by 1.6 mmol/L (95% CI: 0.8-2.3; p < 0.001) between baseline and final visits [51]. These results have been used to guide the design of health care and services aspects of the national policy for diabetes and hypertension.

Involvement of traditional healers (who constitute an important parallel traditional health care system to the biomedical health) may have a beneficial effect in the fight against chronic disease [52]. Indeed, results from a CamBoD qualitative study indicate that training of traditional healers in the fight against diabetes led them to refer their patients for blood glucose tests at biomedical health facilities more often, desist from scarifying patients with diabetes, and educate their patients, peers and other people in their communities about diabetes [52].

A National Cancer Committee based in the Ministry of Public Health exists since 1990, and has been organizing periodic screenings/sensitization campaigns for breast, prostate and cervical cancers (two or three times a year) [48]. In 2002, a national cancer control policy or program was adopted [48]. However, data on the activities and achievements of this program is scarce.

The Cameroonian government has started to prioritize chronic non-communicable diseases in his health agenda. However, this is mostly happening for diabetes, probably because the vast majority of the available evidence to formulate policies originates from the local diabetes research community.

### Fiscal and regulatory interventions

Tobacco control policies are not prioritized in Cameroon. Although there is a national tobacco control program, no data on the trends in tobacco exists, to evaluate such a program. A number of tobacco control measures theoretically exist in Cameroon, including some limited smoke-free provisions/legislation (current national smoke free legislation covers healthcare, educational and government facilities), an advertisement ban and some labeling requirements. However, implementation and enforcement of these measures are still problematic. It is usual not to have health warnings on cigarette packages and advertisements in Cameroon. Though Cameroon has ratified the WHO's Framework Convention on Tobacco Control (FCTC), none of the existing measures is FCTC-compliant [53,54]. The national tobacco control and taxation bill do not include all aspects of FCTC. There is a need to render the tobacco control program more effective, enforce the legislation that prohibits smoking in all indoor public places, close the loopholes that allow the tobacco industry to continue to advertise freely; and increase public awareness of the health hazards associated with cigarettes and other tobacco products; as well as restrict young persons' access to cigarettes.

To date, there is no national nutritional policy in Cameroon. Although there is a law on food labeling, which mandate that information on the nutritional value of foods, microbial content and additives be clearly displayed on packaging, it is too recent to have been effectively implemented [55]. However, any such implementation should account for the functional illiteracy of a fraction of the Cameroonian population (32%) [1]. The food sector needs formal regulation that would mainly aim at foreign multinational food companies that are the main suppliers of snacks sold locally and food rich in additives and trans-fats.

There are also no regulations regarding the nutritional content of meals served in schools, or the amount of physical education required in schools, even if theoretically physical activity is part of the curriculum in secondary schools. It is important to note that very few schools may actually have on-campus catering facilities.

### Community interventions

Nation-wide campaigns to promote physical activity, good nutrition, and tobacco control do not exist. Sporadic activities are often organized around particular events such as the world diabetes day, world heart day, etc... However, studies in the field of diabetes suggest that awareness campaigns for the prevention of chronic diseases can achieve very good results. Indeed, a 4-year long intensive health promotions activity within the framework of the CAMBoD pilot study, in a semi-urban population, raised the level of diabetes awareness among adult Cameroonians [56]. These health promotion activities used all conventional (mass media, health facilities, distribution of health education materials) and non-conventional (meetings in market places, in churches/mosques, health education activities in schools, drama on diabetes in TV/radios) methods. These findings lay the groundwork for further studies and provide evidence policy making.

### Role of the private sector

In Cameroon, the private sector has traditionally been an under-utilized partner for health promotion, primary and secondary prevention of chronic diseases, and cost-effective management. It is unclear how much the local and multinational companies installed in the country spend in the health of their employees. The concept of health insurance is relatively new in the Cameroonian environment. It would therefore be difficult to conceive of local prevention strategies that revolve around health insurance, such as wellness program initiatives and benefits in which health-seeking behaviors such as health-risk assessments, gym membership, purchase of healthy foods, opportunistic screening, chronic disease management programs, and worksite wellness interventions, would be fully or partially subsidized and in some cases incentivized.

Within the framework of the CAMBoD project of sentinel surveillance implemented in four sites, a public-private partnership was established, involving a number of pharmaceutical firms to supply diagnostic equipment for the early detection of diabetes and support the provision of training for medical and paramedical staff in diabetes care [47]. This initiative has been contributing to improvements in diabetes care. Also, the Cameroonian Ministry of Public Health signed an agreement with one pharmaceutical company in 2006, to ensure a reduction in the price of insulin. Partnerships were also sought with non-profit organizations. Rotary Clubs were invited to back a pilot project to offer free comprehensive care for children with type 1 diabetes in Cameroon [47]. The Cameroon Ministry of Public Health assumed responsibility for the provision of care at the sentinel sites that provided research data under CAMBoD. The project is currently been extended into other provinces, with the objective to cover all of Cameroon's ten provinces [47].

Additional efforts are needed to encourage further engagement of the private sector in activities directed at the prevention and management of all chronic diseases. This could be done through corporate social investment activities, e.g., the provision of dedicated research and training funding, the implementation or support of school- or community-based programs promoting physical activity, healthy eating, and tobacco control, as well a social marketing campaigns focusing on healthy choices and health-seeking behaviors.

## Conclusion

The burden of non-communicable chronic diseases is fast increasing in Cameroon, probably because of the rise in the prevalence of risk factors in the population. Local authorities have been making efforts to set up policies for control and prevention of these conditions, especially for diabetes and hypertension. However, a lot remains to be done in developing a more comprehensive surveillance system to guide policy. There is a need to strengthen vital registration, nationally representative population-based surveys and health system information systems to provide comprehensive information on risk factors for chronic diseases, in order to design and review effective programs. The Cameroonian government can respond by better embracing the demands of the dual burdens of acute and chronic diseases, and create multi-sectoral, integrated programs for prevention, early diagnosis, and cost effective treatment to optimize the use of limited resources. An integrated approach to health care that accounts for both infectious and chronic diseases appears to be the way forward. Another aspect that needs to be addressed is the training of health professional, in which public health aspects of the prevention and care for non-communicable chronic diseases need to be incorporated.

## Competing interests

The authors declare that they have no competing interests.

## Authors' contributions

JBE and APK drafted the manuscript. All authors read and approved the final manuscript. JBE is the paper guarantor.
